# The cluster analysis of traditional Chinese medicine authenticity identification technique assisted by chemometrics

**DOI:** 10.1016/j.heliyon.2024.e37479

**Published:** 2024-09-05

**Authors:** Yunxia Bai, Huiwen Zhang

**Affiliations:** a College of Computer Science and Technology, Baotou Medical College, Baotou, 014040, China; b College of Pharmacy, Inner Mongolia Medical University, Hohhot, 010110, China

**Keywords:** Chemometrics, Cluster analysis, Traditional Chinese medicine authenticity identification technique, Quality control

## Abstract

This study explore the authenticity identification technique of traditional Chinese medicine (TCM) using chemometrics in conjunction with cluster analysis. A clustering Gaussian mixture model was constructed and applied for the data clustering analysis of four types of TCM. Chemical measurements combined with discrete wavelet transform (DWT), Fourier transform infrared spectroscopy (FTIR), and Fourier self-deconvolution (FSD) were utilized for the detailed differentiation of Bupleurum scorzonerifolium, Bupleurum yinchowense, Bupleurum marginatum, and Bupleurum smithii Wolff var. parvifolium. Differences in the attenuated total reflection-FTIR (ATR-FTIR) spectra among the four TCMs were observed. Utilizing clustering algorithms, the one-dimensional DWT of the infrared spectra of samples was employed for the authentication of Chinese herbal medicines. The model demonstrates optimal performance throughout 2000 rounds of network training. The accuracy (88.6 %), sensitivity (86.5 %), and specificity (82.7 %) of the model constructed in this study significantly surpassed those of the CNN model: accuracy (67.7 %), sensitivity (70.4 %), and specificity (68.5 %) (*P* < 0.05). By setting the cluster size *K* = 5 and the number of Gaussian mixture model components to 5, the model effectively fits the actual number of categories within the dataset. Infrared spectroscopy analysis revealed distinct carbon-oxygen stretching vibration absorption peaks between 1025 and 1200 cm^−1^ for Bupleurum scorzonerifolium, Bupleurum yinchowense, Bupleurum marginatum, and Bupleurum smithii Wolff var. parvifolium, indicating strong absorption peaks of carbohydrates. A comprehensive structural information analysis revealed a similarity of above 0.982 among the four types of TCM. Combined with chemometrics and intelligent algorithm-based cluster analysis, successful and accurate authentication of TCM authenticity was achieved, providing an effective methodology for quality control in TCM.

## 1 Introduction

### 1.1 Research background and motivations

Traditional Chinese medicine (TCM) plays a significant role in maintaining public health. Authenticity identification of TCM has been a crucial issue within the field of TCM, acting as a pivotal aspect in quality control of TCM products [1]. With the widespread usage of TCM in the market, the issue of authenticity not only affects the effectiveness of patient treatment but also impacts the credibility and economic interests of the entire TCM industry [2,3]. Hence, developing an efficient, accurate, and reliable authenticity identification technique for TCM has become an urgent concern.

Chemometrics is a discipline that explores the quantitative relationships among substances, focusing on the quantitative connections among various substances in chemical reactions [4,5]. It's a mathematical tool and technique to analyze data from multiple perspectives, enabling the identification of complex samples and the study of multidimensional datasets at a molecular level [6,7]. Chemometric quantities encompass concepts such as moles, chemical equations, limiting reagents, equilibrium constants, and thermochemistry of reactions [8,9]. In TCM authentication, chemometrics primarily serves for comprehensive analysis and mathematical model predictions. This approach enables the analysis of spectral data and information about the components of TCM samples, enhancing the accuracy of TCM authentication. Additionally, it aids in reducing subjective judgment errors and drives innovation in TCM authentication technology [10,11]. Cluster analysis is a technique that categorizes similar objects within a dataset into the same group. Its objective is to divide the objects in the dataset into several categories or clusters based on the analysis of their similarities, without prior knowledge of category information [12]. In fields such as bioinformatics, medicine, and social sciences, cluster analysis is widely applied to explore patterns, relationships, and structures within data [13,14]. Chemometrics employs mathematical and statistical approaches to handle and interpret chemical data. When combined with cluster analysis, it facilitates better utilization of information within datasets to uncover patterns and regularities [15,16]. The integration of chemometrics and cluster analysis is employed for pattern recognition, aiming to identify similarities or differences among samples by analyzing patterns within the data [17]. In the authentication of TCM, the amalgamation of chemometrics and cluster analysis is utilized for classification and differentiation of various herbal samples. By comparing their compositional features, similar samples are clustered together, aiding in the rapid and accurate differentiation of different TCMs.

In the authentication of TCM, various techniques such as mass spectrometry and chromatography are commonly employed for multimodal analysis [18]. Cluster analysis integrates these diverse data sources to comprehensively explore the compositional characteristics of Chinese herbal samples. Additionally, fingerprint analysis is a prevalent method in herbal authentication research [19,20]. Tarapoulouzi et al. (2022) [21] suggested that chemometrics introduces novel functionalities in data analysis and the management of complex matrices. The efficacy of herbal products relies on the content of their active ingredients, which can vary significantly across different concentrations. Rebiai et al. (2022) [22] correlated chemometric approaches with the identification of plant chemical diversity based on critical bioactive compounds across several closely related species. Beyond predicting active ingredients, these techniques are utilized to distinguish between varieties and hybrids through quantitative analysis methodologies. Chemometric methodologies serve as a crucial and robust tool for quality control and authentication across various herbal remedies. Wulandari et al. (2022) [23] indicated that employing chemometric methodologies allows for a comprehensive quality control assessment throughout pharmaceutical formulation production processes. Compared to traditional methodologies, chemometrics presents several advantages, such as non-destructiveness, direct analysis of samples without extraction, elimination of stability studies, and cost-effectiveness. Tian et al. (2020) [24] established a DNA fingerprinting method using STR profiling to create DNA fingerprints of bear bile powder and its adulterants, achieving differentiation between authentic bear bile and its adulterated forms. Each species possesses distinct STR fingerprints, enabling the differentiation between different species and the authentication of authenticity. Liu et al. (2023) [25] discussed the application of modern analytical techniques combined with chemometrics in the identification of Chinese medicinal material sources, processing methodologies, cultivation techniques, and adulteration, providing insights into TCM identification. Liu et al. (2022) [26] analyzed the research progress in detecting herbal medicine fraud using modern analytical techniques combined with chemometrics, offering reference value for herbal medicine efficacy identification. Scholars have applied metabolic fingerprint analysis, involving nuclear magnetic resonance spectroscopy and liquid chromatography, to herbal product identification [27]. This metabolic fingerprint analysis provides an efficient and reliable method for differentiating authentic TCMs. Yue et al. (2021) [28] employed chemometrics to analyze the medicinal value of ginseng, while Long et al. (2022) [29] used a multimodal fingerprint combined with chemometric methodologies to establish a simple and effective approach for discriminating between different sources of Cortex Fraxini. Xiong et al. (2019) [30] analyzed the fingerprint of active ingredients from animal bile sources in TCM using high-performance liquid chromatography coupled with evaporative light scattering detection (HPLC-ELSD) and chemometrics. Gong et al. (2017) [31] elaborated on the role of chemometrics, multi-wavelength fusion spectra, and average linear quantitative fingerprinting in controlling the quality of TCM and medicinal formulations. By combining chemometrics with multiple spectral analysis techniques, the quality of TCM and medicinal formulations, including the evaluation of antioxidant activity, can be comprehensively and practically controlled. Chen et al. (2011) [32] explored the quality control of TCM using ultra-high-performance liquid chromatography (UHPLC), which enables researchers to conduct quality control more rapidly and sensitively, ensuring that TCM products meet the required standards. Gaião Calixto et al. (2023) [33] analyzed the application trends of chemometric pattern recognition techniques in the analysis of medicinal plants, emphasizing the prospects and value of this technology in medicinal plant research. The aforementioned studies demonstrate the diverse applications of chemometrics in the field of TCM, offering advanced technical means and methodologies for TCM research and quality control. These techniques, based on comprehensive analyses of multidimensional data, not only provide a more complete reflection of the complex components of TCM but also enhance the objectivity and scientific rigor of identification. Chemometrics, by establishing mathematical models, reveals potential relationships among various components in TCM, presenting new avenues for authenticity determination. Meanwhile, cluster analysis further enhances the accuracy of TCM authentication by categorizing TCM samples into distinct groups.

### 1.2 Research objectives

**Fig. 1:**
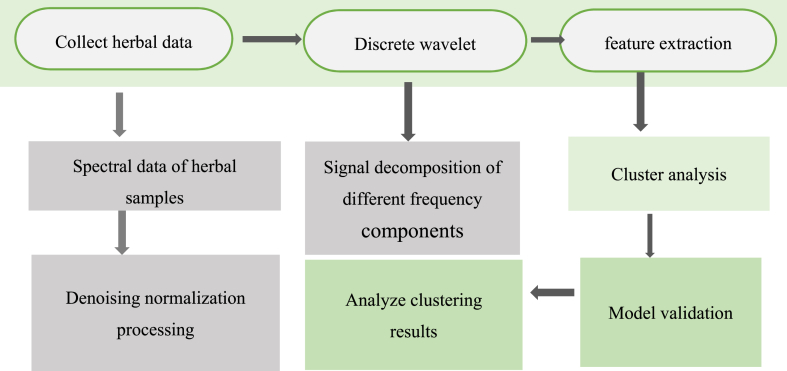
Flowchart of the herbal processing procedure in this study.

Traditional methodologies for authenticating Chinese herbal medicine are prone to subjective judgments, which can lead to potential interference from individual biases. However, integrating chemometrics with cluster analysis techniques mitigates subjective judgment errors by employing data-driven analysis, thereby enhancing the objectivity of authentication. The application of chemometrics allows for comprehensive and systematic determination of multiple components within Chinese medicine, which may improve the accuracy and credibility of authentication. By combining chemometrics and cluster analysis, multiple indicators can be considered simultaneously, facilitating a holistic evaluation of the authenticity of Chinese medicine and aiding in uncovering underlying patterns within complex systems. This study explores a novel approach to authenticating Chinese herbal medicine by integrating advanced chemometrics and cluster analysis techniques to analyze molecular characteristics. This integration offers a new and efficient solution for authenticating Chinese herbal medicine, thereby advancing quality control and supporting industrial development. Additionally, it aims to foster innovative approaches in the field of herbal authentication, driving the overall advancement of this domain. The flowchart of the current study is depicted in Fig. 1.

## 2 Research methodology

### 2.1 Chemometrics

The core of chemometrics involves translating complex chemical information from samples into mathematical models. In the analysis of authenticity in TCM, chemometrics employs two key methodologies: Partial Least Squares (PLS) regression and Principal Component Analysis (PCA). These methodologies are used for classification, clustering, and variance analysis of samples by processing mathematical models.

#### 2.1.1 The following code implements PLS regression

PLS regression is a multivariate statistical method designed for building predictive models. It functions by establishing a linear regression model that maximizes covariance between the dependent and independent variables, extracting latent variables to predict the response variable. PLS is particularly well-suited for datasets with high correlation and multicollinearity among independent variables. In this study, PLS is used to analyze the relationship between infrared spectral data and the chemical composition of Chinese medicinal materials. By employing the PLS model, complex spectral data are simplified into a few latent variables, which are then used to predict sample categories, enabling the identification of authenticity and variety in Chinese medicinal materials.

#### 2.1.2 The principles of the two algorithms

PCA is a dimensionality reduction technique that transforms high-dimensional data into lower-dimensional data while retaining as much of the original data variance as possible through linear transformations. By identifying the principal components, which are directions of maximum variance in the data, PCA projects the data onto these components, achieving dimensionality reduction and feature extraction. In this study, PCA is used to reduce the dimensionality of infrared spectral data. Through PCA, high-dimensional spectral data are transformed into several principal components that explain a significant portion of the data variance. The results of PCA enhance our understanding of the differences between various Chinese herbal samples and provide a foundation for further cluster analysis.

### 2.2 Cluster analysis

#### 2.2.1 Algorithm and model

The clustering algorithm primarily comprises three components.

i. Establishing a matrix A to represent the sample set;
ii. Computing K eigenvalues and eigenvectors within A;

Initially, the original sample data are mapped to a one-dimensional space (k = 1) within a multi-dimensional space. This space, denoted as B’, is generated from K orthogonal vectors in a k-dimensional space.

iii. Utilizing the rows of the k-dimensional subspace A′ as a new data representation for the samples, followed by clustering of the samples.

The objective function operates within a one-dimensional space to optimally partition the data based on the principle of the objective function. It iteratively partitions the delineated subgraphs. Subsequently, the clustering algorithm functions within the k-dimensional space, establishing a broad framework for the algorithm's execution.

The objective function equation is A = D, see equation (1):

(1) $$A=D^{-\frac{1}{2}}LD^{-\frac{1}{2}}$$

in the equation, *D* is a diagonal matrix, see equation (2):

(2) $$W={\left[W_{ij}\right]}_{n\times n}$$

*W*_*ij*_ represents the weights of connecting vertices *i* and *j*, and the Laplacian matrix of the graph is *L* = *D*-*W*.

Structural clustering utilizes a Gaussian mixture model, a probabilistic framework based on multiple Gaussian probability density functions. The corresponding equation (3) is as follows:

(3) $$H_{n}\left(y|\delta \right)=\sum_{k=1}^{k}\beta_{k}\tau \left(y|\delta_{k}\right)$$

$\beta_{k}$ represents the weight of each mixture component, subject to the constraint, $\sum_{k=1}^{k}\beta_{k}=1:\tau \left(y|\delta_{k}\right)$ denotes the Gaussian distribution density function, where $\delta_{k}=$ ($\mu_{k,}\mu_{k}²$).

The number of Gaussian components *K* is determined by minimizing the Bayesian information criterion, which assesses the fit of the Gaussian mixture model to the observed data. The training data is directly obtained from simulated raw output, categorizing some irregular aggregates as “other” classes. For irregular local results, the reconstruction loss should exceed that of aggregates with regular structures. The Gaussian model partitions the entire dataset by testing different components, calculating the maximum cluster loss *HK* when the component number is *K*, which represents the loss of irregular aggregates. equation (4) is as follows:

(4) $$H_{K}=\underset{2\leq k\leq K}{\mathrm{MAX}}\left(H_{p,K}+H_{w,K}\right)$$

$H_{p,K}$ and $H_{w,K}$ represent the reconstruction loss and weighted reconstruction loss of cluster *K*, which can be computed for different *K* values to obtain the optimal number of components, with the corresponding *K* obtained at the inflection point.

**Fig. 2:**
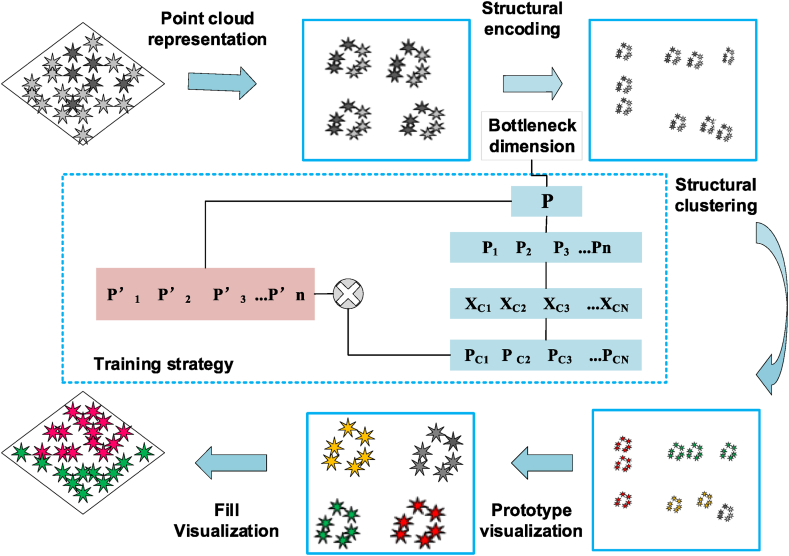
Flowchart depicting the process of local structure identification and training strategy for the model.

Utilizing the Gaussian model for structural clustering and employing autoencoders for dimensionality reduction, Fig. 2 illustrates the workflow for local structure recognition. Initially, the point cloud undergoes representation, followed by structural encoding and bottleneck dimensionality. Subsequently, structural clustering is performed, concluding with prototype visualization and visual fillings. The bottleneck dimension, denoted as *C*, is a critical factor in feature learning and network training; a dimension that is too small may lead to underfitting, while an excessively large dimension could result in overfitting. In this study, an online data augmentation network that randomly rotates point clouds at selected stages is employed. *P*’ represents the reconstructed point cloud, for which the loss function is computed for each output in relation to the corresponding input, followed by the backpropagation process.

#### 2.2.2 Loss function

The loss function quantifies the discrepancy between the predicted values and the ground truth, aiming to minimize this error through network training. equation (5) below represents the loss function employed in this study:

(5) $$W_{TOTAL}=W_{P}+W_{r}+W_{n}+W_{c}+W_{e}$$

in the equation, *Wp* represents the reconstruction loss; *Wr* represents the center-weighted reconstruction loss; *Wn* represents the overlap loss; *Wc* represents the cross-rotation-consistent loss; and *We* is the regularization loss.

This work adopts the Chamfer distance as the reconstruction loss function, defined for two sets of points, *P*_1_ and *P*_2_, of the same size. The Chamfer distance equation (6) is as follows:

(6) $$B_{c}\left(N_{1}，N_{2}\right)=\frac{1}{\left|N_{1}\right|}\sum_{N_{i}\in N_{1}}\lim_{N_{j}\in N_{1}}\left|{\left|N_{i}-N_{j}\right|}_{2}^{2}+\frac{1}{\left|N_{1}\right|}\right|{\sum_{N_{i}\in N_{1}}\left|N_{i}-N_{j}\right|}_{2}^{2}$$

where, |*N*| represents the size of set *N*.

The center-weighted reconstruction loss function equally treats the mappings from input to output and from output to input. The aggregates in this study are defined based on the proximity to the central particles. After readjusting the reconstruction loss function, the asymmetric form of the Chamfer distance is represented by Equation (3).

The center weighted reconstruction loss *Wr* equation (7) is as follows:

(7) $$W_{r}=W_{P}+Q_{r}\left[\lambda cwDcw\left(N,N^{\prime }\right)\right]+\lambda cwDcw\left(N,N^{\prime }\right)$$

*Qr*, *λ*_*CW*_, and *D*_*CW*_ indicate weight coefficients.

*W*_*n*_ represents overlap loss, see equation (8) below:

(8) $$W_{n}=\frac{Qn}{\left|M_{n}\right|}\sum_{N_{j}\in N_{1}}\sum_{N_{j}\in N}E\left(N_{i},N_{j}\right)\;j>i$$

*E* (*N*_*i*_’, *N*_*j*_’) is the overlap function of particles *i* and *j*, *Q*_*n*_ is the number of particle pairs, and *M*_*n*_ is the weight coefficient of the loss function.

*W*_*c*_ represents cross rotation consensus loss.*W*_*e*_ is the regularization loss, see equation (9) below:

(9) $$W_{e}=Rr\sum_{N_{j}\in N}{\left|\left|\varphi \right|\right|}^{2}$$

*Rr* is the weight coefficient of the loss function, and φ is the training parameter of the network.

## 3 Experimental design and performance evaluation

(1) Discrete wavelet transform (DWT)

DWT is a signal processing technique that analyzes the frequency-domain characteristics of a signal by decomposing it into wavelet basis functions at different scales. Widely applied in signal processing, image processing, and data compression, DWT provides localized information in both time and frequency domains, enabling signal analysis across various scales.

(2) Fourier transform infrared spectroscopy (FTIR)

FTIR provides comprehensive chemical composition data of herbal samples, aiding in the analysis of various organic constituents present in traditional medicines. FTIR spectra help identify typical functional groups within herbal components and their molecular structures. By comparing spectral features among different herbal medicines, FTIR assists in authenticating and detecting moisture content in herbal products, ensuring compliance with storage and usage standards.

(3) Fourier self-deconvolution (FSD)

FSD enhances the resolution of spectra. Due to the complexity of herbal materials, employing FSD effectively facilitates the observation of overlapping absorption peaks in samples. Coupling this technique with DWT for image analysis and processing aids in reducing and optimizing data, thereby enabling accurate classification of authenticity in herbal medicines.

### 3.1 Experimental materials (or datasets collection)

#### 3.1.1 Experimental materials

Bupleurum scorzonerifolium refers to the dried roots of the umbrella plant *Bupleurum scorzonerifolium Willd*; Bupleurum yinchowense refers to the dried roots of the umbrella plant *Bupleurum yinchowense Shan et Y. Li*. Bupleurum marginatum represents the dried whole herb of *Bupleurum marginatum Wall.ex DC*, a member of the Umbelliferae family. Bupleurum smithii Wolff var. parvifolium pertains to the dried roots of the Umbelliferae plant *Bupleurum smithii Wolff* var. *parvifolium Shan et Y. Li*. After collection, all samples are air-dried indoors and further dehydrated in an oven at 60 °C for 72 h. Ten randomly selected root samples are pulverized using a grinder into fine powder, weighed at 10.0 mg, and set aside for further analysis.

#### 3.1.2 Measurement methodologies

The attenuated total reflection-FTIR (ATR-FTIR) spectroscopic technique was employed to analyze surface composition and structural information of materials. In this study, the diamond ATR accessory was placed horizontally in the sample compartment of the FTIR instrument as per instrument requirements, ensuring a consistent contact area between the material powder and the device. Mechanical calibration pressure was kept constant, and a background scan was conducted before each sample measurement. Each measurement was repeated three times. The obtained infrared spectra underwent baseline correction. The ATR-FTIR spectra were processed using the OMNIC E.S.P5.1 software to acquire FSD infrared spectra. Subsequently, a clustering algorithm was applied to analyze the one-dimensional continuous wavelet transforms of the sample's infrared spectra, aiming to observe differences in the ATR-FTIR spectra and thereby authenticate the authenticity of TCM.

#### 3.1.3 Performance evaluation metrics

A reasonable assessment of performance metrics effectively evaluates the performance of algorithms. Ultrasound image cross-sections were evaluated as a binary classification problem, with model-predicted categories divided into true negatives (TN), false positives (FP), true positives (TP), and false negatives (FN). The accuracy calculation is shown in Equation (10), where higher classification accuracy indicates better algorithm performance. Precision measures the proportion of true positive samples among all samples predicted as positive, while specificity, also known as recall, represents the proportion of true positive samples among all positive samples. The harmonic mean of recall and precision is higher when both recall and precision are high; if one is low, the harmonic mean decreases, approaching the lower value, as illustrated in equations (10), (11), (12), (13).

(10) $$A\text{ccuracy}=\frac{TP+FN}{TP+FP+TN+FN}$$

(11) $$\mathrm{Pr}\text{ecision}=\frac{TP}{TP+FP}$$

(12) $$\text{Specificity}=\frac{TN}{FP+TN}$$

(13) $$\text{Sensitivity}=\frac{TP}{TP+FN}$$

### 3.2 Experimental environment

The network was trained using the LJSpeech publicly available dataset, which comprises 131,000 recorded data entries. The experimental hardware setup included 128 GB of RAM, two NVIDIA 1080 Ti GPUs, and an Intel Xeon® Silver 4110 CPU @ 2.10 GHz with 32 cores, running on the Ubuntu 16.04 operating system. PyTorch 1.2.2 served as the deep learning framework, utilizing Python 3.6 as the development language.

### 3.3 Parameter setting

#### 3.3.1 Training network results

**Fig. 3:**
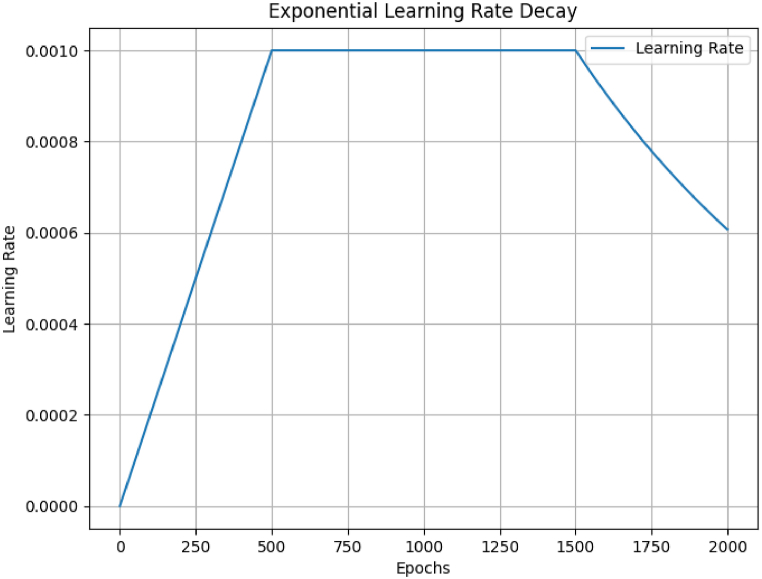
Network training results.

The Adam optimization methodology [34] was employed for training the network over 2000 epochs. The prediction mechanism was initiated after the first 500 epochs, during which the learning rate gradually increased from 0 to 0.001. Subsequently, a learning rate exponential decay commenced at 1500 epochs (Fig. 3).

#### 3.3.2 Dataset parameter selection results

**Fig. 4:**
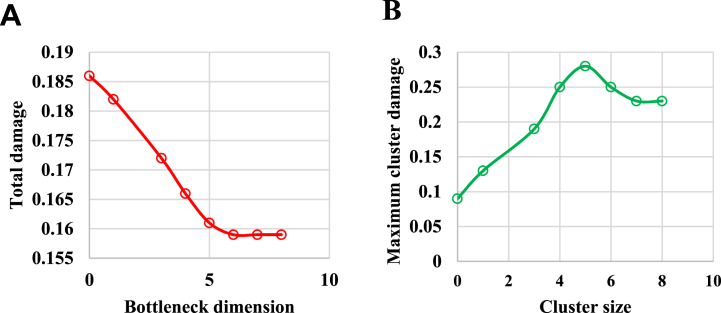
Results of dataset parameter selection. Note: (A) Curve of bottleneck dimension *C*; (B) Curve of cluster size *K*.

The dataset comprising LJ particles was selected to validate the algorithm. Initially, raw data were preprocessed to fit the algorithm model in this study, with the particle radius set at 0.5. The weights of the damage functions were specified as follows: γp = 0.1, γ0 = 0.001, and γc = 0.001, with a truncation radius of 1 in the central weighted damage. Fig. 4A illustrates the curve of bottleneck dimensionality C, showing a decreasing trend in model loss. A turning point is observed at C = 5, indicating that the network model has acquired sufficient information. Fig. 4B presents the loss curves for different cluster sizes (K), where the Y-axis represents the losses obtained for various cluster sizes. The curves reveal that loss gradually decreases with an increase in cluster size. Notably, the minimum loss value is observed at K = 5, indicating optimal model performance at this cluster size. Therefore, K = 5 is selected as the optimal cluster size for the dataset.

#### 3.3.3 Performance metric results

**Fig. 5:**
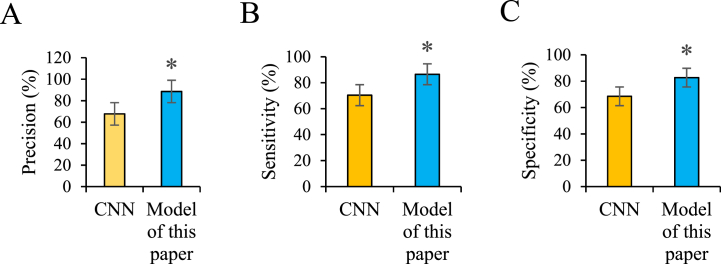
Performance metric results. (Note: (A) Precision; (B) Sensitivity; (C) Specificity. *P < 0.05.).

In Fig. 5A, B, 5C and 5D, the model constructed in this study are compared with the classical CNN. The accuracy rates are 67.7 % and 88.6 % respectively, sensitivity rates are 70.4 % and 86.5 % respectively, and specificity rates are 68.5 % and 82.7 % respectively. The accuracy, sensitivity, and specificity of the model in this study are significantly higher than those of the CNN model (*P* < 0.05).

#### 3.3.4 Dataset training curve

**Fig. 6:**
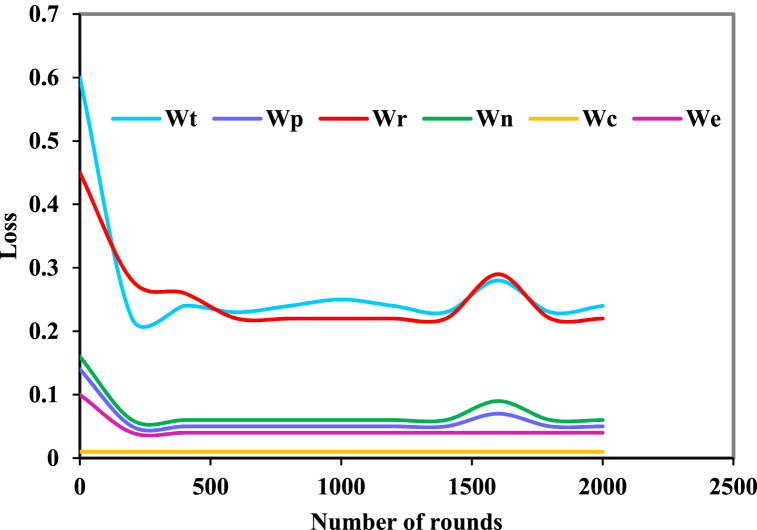
Training curve of the dataset. (Note: *Wp* represents reconstruction loss; *Wr* represents weighted center reconstruction loss; *Wn* represents overlap loss; *Wc* represents cross-rotation consistency loss; *We* represents regularization loss.).

Fig. 6 illustrates the training history of the training set within the model. Initially, a declining trend in total loss is observed, followed by a stabilization phase. The cross-rotation consistency loss and overlap loss occupy a relatively smaller portion within the total loss, hence appearing towards the bottom of the network training learning curve.

### 3.4 Performance evaluation

#### 3.4.1 FTIR analysis of four TCM samples

**Fig. 7:**
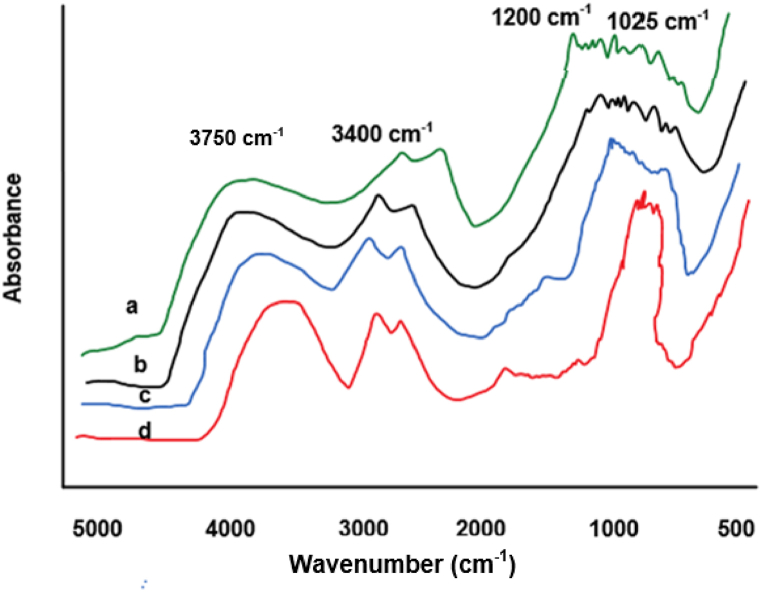
FTIR of Bupleurum scorzonerifolium and its counterfeit. (Note: A: Bupleurum scorzonerifolium; B: Bupleurum yinchowense; C: Bupleurum marginatum; D: Bupleurum smithii Wolff var. parvifolium).

From Fig. 7A, B, 7C and 7D, they are evident that Bupleurum scorzonerifolium, Bupleurum yinchowense, Bupleurum marginatum, and Bupleurum smithii Wolff var. parvifolium exhibit distinct absorption peaks associated with carbon-oxygen stretching vibrations within the range of 1025∼1200 cm^−1^. These peaks, characteristic of strong carbohydrate absorption, indicate a significant presence of cellulose and other carbohydrates in these four TCM samples, which belong to the same botanical family and genus. Additionally, absorption peaks at 3400 cm⁻^1^ were attributed to hydroxyl stretching vibrations. Although the FTIR spectra reveal some variations, they are not substantial. The absorption peak at 3750 cm⁻^1^ is ascribed to the stretching vibration of hydroxyl groups, indicating the presence of hydroxyl functional groups in these herbal samples. Such groups are commonly associated with glucose, carbohydrates, and other oxygen-containing organic compounds, suggesting the presence of polysaccharides. While specific absorption peak characteristics may vary among samples, the consistent presence of the hydroxyl stretching vibration absorption peak at 3750 cm⁻^1^ supports the presence of polysaccharide compounds in these samples. To further validate the authenticity of these TCMs, a model employing clustering algorithms and discrete wavelet transform for classification and authentication is used. In signal processing, DWT decomposes the signal into components of different frequencies, facilitating clustering analysis of these components to identify specific signal patterns.

#### 3.4.2 FSD analysis results

**Fig. 8:**
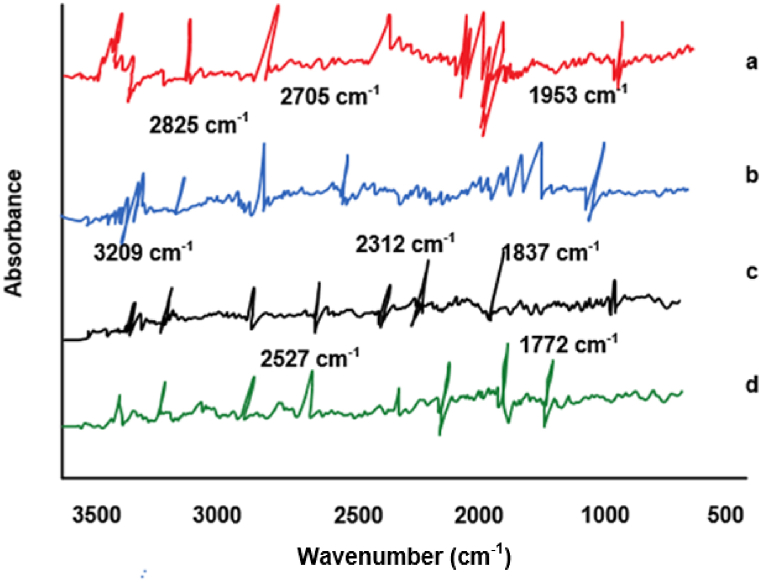
FSD of Bupleurum scorzonerifolium and its counterfeit. (Note: A: Bupleurum scorzonerifolium; B: Bupleurum yinchowense; C: Bupleurum marginatum; D: Bupleurum smithii Wolff var. parvifolium).

Further analysis using FSD reveals the presence of both saturated and unsaturated C-H bonds within the absorption peak range of 2000–4000 cm⁻^1^ among the four types of TCM. Additionally, OH and NH2 groups are identified within this range. Fourier self-deconvolution infrared spectra (FSD-IR) analysis is utilized to determine the phylogenetic relationships among the samples based on the FSD-IR results (Fig. 8A, B, 8C and 8D).

#### 3.4.3 DWT results of four TCM samples

**Fig. 9:**
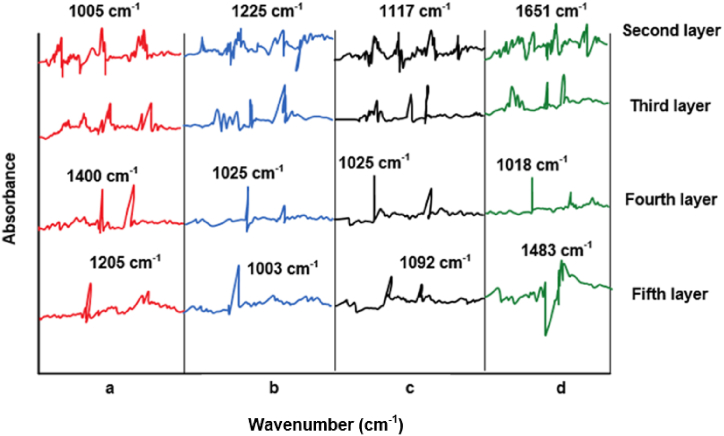
DWT of Bupleurum scorzonerifolium and its counterfeit. (Note: A: Bupleurum scorzonerifolium; B: Bupleurum yinchowense; C: Bupleurum marginatum; D: Bupleurum smithii Wolff var. parvifolium).

The FSD-IR method effectively distinguishes Bupleurum scorzonerifolium from Bupleurum smithii Wolff var. parvifolium, but encounters difficulties in differentiating Bupleurum yinchowense from Bupleurum marginatum. Further analysis involves employing the spectral characteristics of the DWT signals in conjunction with clustering algorithms to assess data, evaluating the efficacy of signal decomposition at various resolutions. Optimal wavelet bases are selected based on characteristic peaks from the original spectra to achieve improved signal smoothing. Five layers of ATR-FTIR spectra decomposition are obtained, excluding the first layer due to excessive noise and signal interference, with identification analysis focused on the remaining layers. The DWT data undergo five compression stages, reducing data volume by half after each stage, enhancing resolution, and revealing previously concealed infrared absorptions in the spectra. In layers 2, 3, and 4, the signals from the four samples exhibit similar shapes. However, significant differences are observed in the infrared spectra between Bupleurum yinchowense and Bupleurum marginatum within the 1000 cm^−1^∼1800 cm^−1^ infrared data band range (Fig. 9A, B, 9C and 9D). This demonstrates that the FTIR method, based on DWT combined with clustering algorithms, accurately differentiates Bupleurum scorzonerifolium within the same botanical family and genus, showcasing promising advancements in the precise authentication of TCM.

#### 3.4.4 Cluster analysis

There were observed differences among the ATR-FTIR spectra of the four types of Chinese herbal medicines. By utilizing a clustering Gaussian mixture model, the wavenumber and absorbance data were extracted for statistical clustering analysis. The results of the clustering analysis were then compared with the morphological classification results. A total of twelve ATR-FTIR spectra data points were selected, representing four different types of herbal samples, with three repetitions for each sample. The comparison involved typical absorption peak values and absorbance at different wavenumber ranges. Intelligent algorithms were employed to analyze the data, and the absorption peak values obtained are presented in Table 1.

**Table 1:** The absorbance of the main absorption peaks of four TCM samples.

Wavelength/cm^−1^	a: Bupleurum scorzonerifolium	b: Bupleurum yinchowense	c: Bupleurum marginatum	d: Bupleurum smithii Wolff var. parvifolium
1800	0.0785 ± 0.0001	0.0943 ± 0.0005	0.0864 ± 0.0007	0.0754 ± 0.0008
1767	0.0743 ± 0.0011	0.0865 ± 0.0006	0.0876 ± 0.0006	0.0789 ± 0.0009
1754	0.0704 ± 0.0021	0.0845 ± 0.0001	0.0783 ± 0.0005	0.0821 ± 0.0007
1743	0.0762 ± 0.0031	0.0743 ± 0.0003	0.0704 ± 0.0006	0.0819 ± 0.0006
1684	0.0634 ± 0.0025	0.0765 ± 0.0004	0.0976 ± 0.0001	0.0716 ± 0.0031
1634	0.0543 ± 0.0011	0.0748 ± 0.0007	0.0845 ± 0.0001	0.0689 ± 0.0017
1583	0.0561 ± 0.0001	0.0764 ± 0.0023	0.0743 ± 0.0002	0.0701 ± 0.0023
1567	0.0532 ± 0.0021	0.0809 ± 0.0053	0.0755 ± 0.0001	0.0683 ± 0.0031
1532	0.0521 ± 0.0004	0.0543 ± 0.0062	0.0768 ± 0.0023	0.0604 ± 0.0042
1467	0.0511 ± 0.0065	0.0654 ± 0.0054	0.0784 ± 0.0041	0.0676 ± 0.0045
1483	0.0431 ± 0.0013	0.0704 ± 0.0004	0.0819 ± 0.0032	0.0645 ± 0.0052
1492	0.0378 ± 0.003	0.0762 ± 0.0002	0.0573 ± 0.0002	0.0543 ± 0.0003
1387	0.0354 ± 0.0003	0.0634 ± 0.0017	0.0644 ± 0.0005	0.0555 ± 0.0001
1354	0.0344 ± 0.0004	0.0543 ± 0.0008	0.0734 ± 0.0001	0.0568 ± 0.0001
1347	0.0358 ± 0.0001	0.0661 ± 0.0009	0.0742 ± 0.0006	0.0584 ± 0.0005
1338	0.0348 ± 0.0001	0.0632 ± 0.0004	0.0645 ± 0.0007	0.0519 ± 0.0006
1302	0.0327 ± 0.0001	0.0621 ± 0.0004	0.0547 ± 0.0008	0.0531 ± 0.0073
1298	0.0317 ± 0.0031	0.0711 ± 0.0001	0.0663 ± 0.0009	0.0511 ± 0.0008
1287	0.0291 ± 0.0021	0.0604 ± 0.0001	0.0632 ± 0.0004	0.0534 ± 0.0009
1276	0.0288 ± 0.0043	0.0662 ± 0.0002	0.0523 ± 0.0001	0.0453 ± 0.0004
1265	0.0275 ± 0.0001	0.0634 ± 0.0003	0.0473 ± 0.0003	0.0476 ± 0.0005
1254	0.0263 ± 0.0001	0.0543 ± 0.0005	0.0503 ± 0.0087	0.0587 ± 0.0006
1243	0.0201 ± 0.0051	0.0561 ± 0.0001	0.0487 ± 0.0082	0.0432 ± 0.0007
1232	0.0118 ± 0.0002	0.0532 ± 0.0001	0.0317 ± 0.0051	0.0306 ± 0.0001
1226	0.0154 ± 0.0022	0.0521 ± 0.0023	0.0308 ± 0.0043	0.0381 ± 0.00037
1218	0.0102 ± 0.0032	0.0511 ± 0.0024	0.0312 ± 0.0032	0.0254 ± 0.0065
1209	0.0342 ± 0.0024	0.0223 ± 0.0032	0.0218 ± 0.0051	0.0205 ± 0.0064
1203	0.0106 ± 0.0006	0.0143 ± 0.0007	0.0123 ± 0.0001	0.0116 ± 0.0034

#### 3.4.5 Cluster analysis tree spectrum

**Fig. 10:**
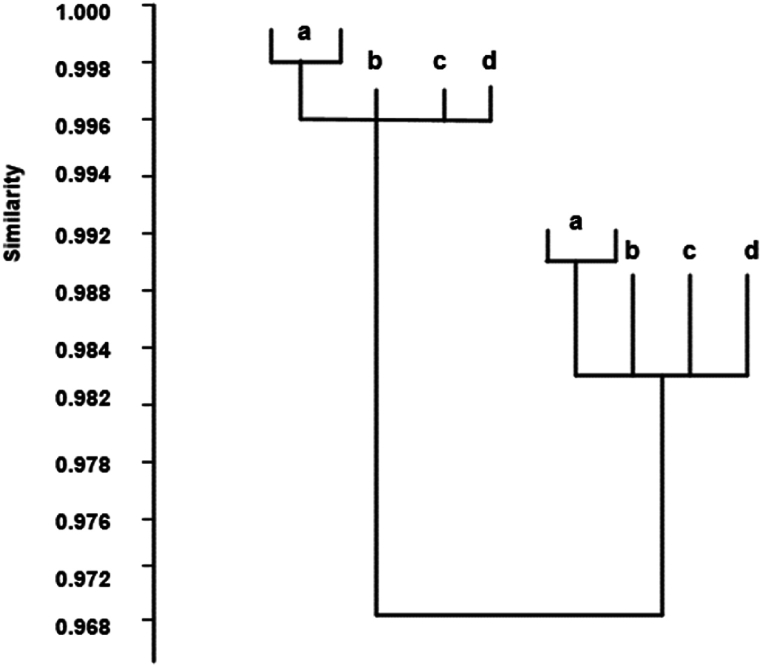
Tree system diagram.

After undergoing analysis using the PAST statistical software package, the ATR-FTIR spectral data were processed through a series of intelligent algorithms, resulting in a dendrogram that displayed the phylogenetic relationships among Bupleurum scorzonerifolium, Bupleurum yinchowense, Bupleurum marginatum, and Bupleurum smithii Wolff var. parvifoliumv (see Fig. 10). The graph demonstrated the structural similarities among the four types of TCM, with a similarity exceeding 0.982. The post-grinding treatment maintained consistency in sample properties aligned with their taxonomic classification for these four medicinal herbs. This highlights how the combination of FTIR with clustering analysis accurately and scientifically differentiated Bupleurum scorzonerifolium from its counterparts, including Bupleurum yinchowense, Bupleurum marginatum, and Bupleurum smithii Wolff var. parvifolium.

### 3.5 Discussion

This discussion provides further insights into the biological and chemical significance of the spectral differences observed among the four types of Bupleurum, facilitating a deeper understanding of their distinctions.

A more detailed analysis of the FTIR spectra and a comprehensive evaluation of how the observed absorption peaks affect the authenticity of Chinese herbal medicine would enrich the research.

The complex chemical composition and diverse pharmacological effects of Chinese herbal medicine present significant challenges in authentication and quality control. Modern analytical techniques have offered new perspectives on this issue. Among these, FTIR is widely used in herbal medicine analysis due to its operational simplicity, rapid analysis, and rich information content [35]. Various methodologies have been employed by researchers to authenticate TCM. For instance, Tan et al. (2015) [36] developed a gas chromatography/mass spectrometry (GC/MS) method based on headspace solvent-free microextraction (HS-SFME) for detecting Angelica sinensis. Zhang et al. (2019) [37] used metabolomics to reveal quality differences between authentic and non-authentic medicinal materials through ultra-high-performance liquid chromatography-quadrupole time-of-flight mass spectrometry (UHPLC-QTOF-MS/MS). Liu et al. (2020) [38] applied electronic eye technology to authenticate the quality of Fritillaria cirrhosa. Yang et al. (2021) [39] used liquid chromatography combined with chemometrics to distinguish between Fritillaria unibracteata and Fritillaria ussuriensis, demonstrating its effectiveness in differentiating Lilium purpurea. These studies underscore the effectiveness of integrating scientific techniques with traditional authentication methods in identifying TCM.Clustering analysis, involving computer-assisted processing of drug data, results in various clustering patterns that aid in the categorization and identification of drugs. Many studies have demonstrated the efficacy of clustering analysis in authenticating TCM. Liu et al. (2023) [40] utilized principal component analysis and clustering analysis to differentiate between Fritillaria chuanensis and Siberian fritillary bulb, showing strong linear correlations within this method. Zhang et al. (2021) [41] employed principal component analysis within clustering analysis for quality control of traditional Chinese medicinal materials, achieving 97.5 % accuracy in the established function model. This study uses intelligent algorithm-based clustering analysis, with results indicating a similarity above 0.982 among the four types of TCM, confirming the efficacy of clustering analysis in authentication. This work integrates chemometrics with clustering analysis for TCM authentication, constructing a Gaussian mixture model for algorithm development and applying it to cluster data for the four types of TCM. After optimized network training, the network performance showed favorable outcomes. In terms of dataset parameter selection, the curve of the bottleneck dimension C indicates that the network model acquired sufficient information at C = 5.

The four types of Bupleurum may contain varying types or levels of chemical constituents, such as flavonoids, triterpene saponins, alkaloids, and others, which may influence the pharmacological effects of the herb. This warrants further investigation and discussion.

FTIR spectroscopy offers valuable structural information on complex TCM systems. Typically, the identification of medicinal herbs involves comparing the types and quantities of functional groups. Infrared spectroscopy facilitates the inference of specific structures corresponding to chemical constituents by analyzing the presentation of hydrogen in various environments. This method aids in distinguishing chemical samples in TCM, including oils and fats.

FTIR technology is a widely utilized analytical method that provides insights into the molecular structure and chemical composition of samples. In the authentication of Chinese herbal medicine, a thorough interpretation of FTIR spectra enhances the understanding of sample differences and assesses their impact on the medicine's authenticity. Detailed analysis of absorption peaks observed in FTIR spectra is crucial, as each peak represents the vibration or stretching of different chemical bonds or functional groups, thus offering information about the sample's composition and structure. By comparing the positions, intensities, and shapes of these absorption peaks across different samples, distinctions between them can be revealed, facilitating authenticity assessment. The chemical composition of the sample can be inferred from the characteristics of the absorption peaks. For instance, absorption peaks within specific wavenumber ranges may indicate the presence of particular chemical constituents such as hydroxyl groups, carbonyl groups, and aromatic compounds. Comparative analysis of absorption peaks helps in assessing the similarity and dissimilarity between samples, thereby aiding in authenticity determination. Factors influencing FTIR spectra, such as sample preparation methods, storage conditions, and sampling positions, must be considered as they may affect the spectra and lead to changes in observed absorption peaks. Therefore, comprehensive consideration of these factors is necessary when interpreting FTIR spectra, with appropriate adjustments and corrections made as needed. in this study, infrared spectroscopy analysis was performed on Bupleurum scorzonerifolium, Bupleurum yinchowense, Bupleurum marginatum, and Bupleurum smithii Wolff var. parvifolium. Combined with DWT and clustering algorithms, successful discrimination among these four herbal medicines was achieved. Within the 1025–1200 cm^-1 region, different peaks corresponding to carbon-oxygen bond stretching vibrations were observed, indicating the presence of carbohydrates in these samples. The intensity and position of these absorption peaks may relate to the chemical composition and structure of the samples. In the 2000–4000 cm^-1 range, absorption peaks associated with saturated and unsaturated C-H bonds, as well as functional groups such as OH and NH2, were observed, providing information about the samples' composition. Comparative analysis of infrared spectra revealed significant differences between the spectra of Bupleurum yinchowense and Bupleurum marginatum and those of other samples. These differences may reflect variations in chemical composition and biological activity among these herbal medicines. For example, different varieties may contain varying types or amounts of active ingredients or exhibit different metabolic pathways and growth environments. Sugars in medicinal herbs are particularly amenable to identification using infrared spectroscopy. This study employed DWT, FTIR, and FSD for detailed identification of Bupleurum scorzonerifolium, Bupleurum yinchowense, Bupleurum marginatum, and Bupleurum smithii Wolff var. parvifolium. Infrared spectroscopy analysis revealed distinct absorption peaks within specific energy bands for these TCM types, notably in the stretching vibrations of carbon-oxygen bonds and hydroxyl groups, indicating significant differences. The combination of DWT and clustering algorithms achieved successful differentiation of the four Bupleurum species, demonstrating high accuracy in herbal medicine identification. The comprehensive structural information analysis showed a high similarity exceeding 0.982 among the four herbal medicines, highlighting the effectiveness of integrating chemometrics with intelligent clustering analysis for precise Traditional Chinese Medicine identification. In summary, the combination of infrared spectroscopy analysis, DWT, and clustering algorithms exhibits high accuracy in distinguishing Bupleurum scorzonerifolium, Bupleurum yinchowense, Bupleurum marginatum, and Bupleurum smithii Wolff var. parvifolium. These differences may be related to the chemical composition and structure of the samples, and further biological or chemical research can elucidate the specific reasons behind these differences, providing a deeper understanding and basis for the authenticity identification of Chinese herbal medicine.

## 4 Conclusions

In this study, the authentication of TCM was conducted using chemometrics combined with clustering analysis. An effective model was achieved through network training by optimizing cluster sizes and varying learning rates. Infrared spectroscopy analysis revealed that four types of TCM exhibited distinct absorption peaks within specific frequency ranges, and these types were accurately distinguished by the clustering algorithm. The integration of chemometrics with intelligent algorithms in clustering analysis provides a precise method for TCM authentication, enhancing the reliability and accuracy of authentication and quality control. This approach broadens the analytical techniques available for TCM authentication.

### 4.1 Research contribution

This study contributes methodologically by advancing TCM authentication techniques through a comprehensive analytical approach. By combining chemometrics with clustering analysis, this research offers an integrated examination of TCM component characteristics, providing a more detailed information set for authenticity determination. It introduces a novel approach to TCM authentication, demonstrating significant advantages over traditional methods due to its multi-index and comprehensive nature. This innovation promises to advance TCM authentication techniques, guiding the field toward a more scientific and systematic direction and offering the TCM industry more reliable quality assurance methods.

### 4.2 Future works and research limitations

Future research should focus on optimizing methodological details. Further refinement of the techniques integrating chemometrics with clustering analysis is necessary to improve the precision and sensitivity of the analyses. Expanding the scope of research samples will validate the applicability of this method across various TCM types and sources, ensuring its generalizability. Further exploration of the interrelationships among TCM components and their impact on authenticity identification will provide theoretical support for refining the method.

Limitations of this study include potential constraints within the sample set, which may not fully represent the complexity of constituent compositions in different TCM types. Expanding the sample range is essential. Additionally, the reliability and accuracy of data acquisition, crucial for the integration of chemometrics with clustering analysis, could impact research outcomes due to data quality issues. Given the relative novelty of this methodology, its stability and feasibility in practical applications require further validation through extensive practical testing. In conclusion, while this study introduces innovative perspectives on TCM authentication, further work is needed to optimize methodologies, expand sample sizes, and explore the underlying mechanisms involved.

## Fundings

This work was supported by the 10.13039/501100004763Natural Science Foundation of Inner Mongolia (No.2022MS08023; 2023QN08049); the Mongolian Medicine Synergy Innovation Center Scientific Research Foundation of Inner Mongolia (No.MYYXTYB202108), and the Key technology Project Foundation of Inner Mongolia (No.2021GG0176); “Flower Bud Plan” Project of Baotou Medical College (No.HLJH202411); Scientific Research Fund Project of Baotou Medical College (BYJJ-KCRH 202418; BYJJ-SZZX 202407); Innovation and Entrepreneurship Training Project for College Students (S202410130023X).

## Data availability statement

Data will be made available on request.

## CRediT authorship contribution statement

**Yunxia Bai:** Writing – original draft, Methodology, Investigation, Formal analysis, Data curation, Conceptualization. **Huiwen Zhang:** Writing – review & editing, Visualization, Validation, Supervision, Software, Resources.

## Declaration of competing interest

The authors declare that they have no known competing financial interests or personal relationships that could have appeared to influence the work reported in this paper.
